# Radioactive Attenuation Using Different Types of Natural Rocks

**DOI:** 10.3390/ma17143462

**Published:** 2024-07-12

**Authors:** S. A. Abd El-Azeem, Nareman M. Harpy

**Affiliations:** 1 Physics Department, College of Sciences and Humanities, Prince Sattam Bin Abdulaziz University, Al-Kharj 11942, Saudi Arabia; 2 Physics Department, Faculty of Women for Arts, Science and Education, Ain Shams University, Cairo 11757, Egypt; 3 Nuclear Materials Authority, P.O. Box 530, El-Maadi, Cairo 11728, Egypt; karem_249@hotmail.com

**Keywords:** shielding, attenuation coefficients, natural rocks, basalt, XCOM program

## Abstract

Humans benefit from nuclear technology, but it also generates nuclear radiation that is bad for both the environment and human health. The serious issue of radiation leakage affects many technological applications. Shielding is required to protect both users and the environment from negative side effects. This work describes the radioactive attenuation properties of some natural rocks, such as claystone, bentonitic claystone, bentonitic shale, sandstone, and basalt using a NaI(Tl) detector. The mass attenuation coefficients $\mu_{m}$ of these rocks at various photon energies, half-value layer (HVL), tenth-value layer (TVL), and mean free path (MFP) were determined. The validation of obtained values of $\mu_{m}$ was carried out against the theoretical calculations from the XCOM program, and the correlation factor and relative deviation between the two methods were evaluated. It was noted that basalt samples exhibit superior shielding parameters when compared to other rock samples. Also, the concentrations of naturally occurring radioactive elements (^238^U, ^226^Ra, ^232^Th, and ^40^K) were measured, allowing for the calculation of environmental hazard indices and assessment of attenuation (%) efficiency for certain natural rocks, such as bentonite, sandstone, and basalt. The results revealed that increasing the thickness of Basalt-AZ from 1.5 cm to 2 cm results in an approximate 11% rise in attenuation percentage, with values reaching 77.12%, 67.2%, 67.65%, and 59.8% for NMA-U, IAEA-Th, IAEA-Ra, and IAEA-K, respectively.

## 1. Introduction

Nuclear technologies and radiation play crucial roles in various fields, including process industries, manufacturing, nuclear medicine, radiology, nuclear power plants, and research centers [1,2,3,4,5,6]. Exposure to elevated radiation levels can have deleterious effects on individuals, the public, and the environment. Radiation protection aims to reduce these risks by protecting the environment and people from the harmful effects of ionizing radiation. Among the radiation protection techniques, shielding is the most effective because it greatly lowers the intensity of incident radiation [7,8,9,10,11]. Lead (Pb) and conventional materials like concrete have historically been used as radiation shields. However, lead is hazardous on its own, and concrete requires thick walls for effective shielding [12,13,14]. Nowadays, the development of novel, user-friendly, and efficient composite shielding materials is crucial. As a result, a great deal of research has been conducted to improve the shielding capacity of different materials [15,16,17,18]. Finding substitute materials that are secure, affordable, and ecologically friendly is therefore urgent. One such material is natural rock, which has shown superior gamma-ray attenuation capabilities compared to concrete [19].

Rocks, omnipresent in our daily surroundings, not only are inexpensive but also find utility in various applications. Examples include limestone, employed in cement production, and bituminous coal, utilized for electric power generation [19]. Basalt, one of the earliest natural rocks utilized by humans, is found extensively across the globe. It encompasses a diverse group of volcanic rocks that originated near the Earth’s crust. A glassy texture is characterized by a non-crystalline, glass-like appearance in basaltic rocks. Glassy basalt typically forms when lava cools very rapidly, preventing the formation of mineral crystals; thus, it is considered an amorphous material. Basalt is characterized by a fine microstructure composed of small crystals and amorphous material due to rapid surface-level volcanic cooling. It exhibits a dark color due to its rich magnesium and iron content. In the current study, the basalts were of an intimate to black type. The specific structure of basalt can vary, primarily based on its formation environment. With an average density ranging from 2.8 to 3.1 g/cm^3^ and a compressive strength between 170 and 200 MPa, basalt stands out as a desirable and cost-effective material for use as aggregate in radiation shielding for radiological facilities [20].

Bentonite, primarily composed of montmorillonite, is versatile clay with additional minerals like feldspar and quartz. The extensive use of this substance can be attributed to its water affinity, large surface area, sheet-like structure, and small crystal size. Bentonite’s corrosion resistance and thermal stability make it a good choice for radiation shielding; it was used in pet waste absorbents, metal casting, papermaking, iron ore palletization, and other applications. These qualities, which are widespread worldwide, highlight how useful it is for such uses [19,20,21,22].

Claystone is a fine-grained sedimentary rock primarily composed of clay-sized particles (less than 1/256 mm in diameter). It forms through the lithification of clay-rich sediments, which typically occurs in low-energy environments such as lakes, deep marine settings, and floodplains. The main minerals found in claystone include various clay minerals like kaolinite, illite, and smectite, along with minor amounts of quartz, feldspar, and other minerals [23].

Sandstone is a clastic sedimentary rock composed mainly of sand-sized mineral particles or rock fragments. It typically consists of quartz, feldspar, and rock fragments bound together by a natural cementing material like silica, calcium carbonate, or iron oxide. The proportion of these components can vary, leading to different types of sandstone [24].

The practical applications of rocks have prompted numerous researchers to investigate the photon-shielding properties of specific natural rock types; for example, Obaid et al. determined that feldspathic basalt, volcanic rock, compact basalt, pink granite, and dolerite are superior to concrete in gamma-ray attenuation [4]. Al-Buriahi et al. studied the radiation-shielding characteristics of natural rocks, including olivine basalt, jet black granite, limestone, sandstone, and dolerite [19]. They found that these rocks provide better shielding than traditional concretes and are comparable to commercial glasses. El-Khatib et al. investigated the gamma-ray-shielding capability of Egyptian bentonite clays enhanced with composites [25]. They concluded that cement-reinforced bentonite materials are effective for gamma-radiation protection and can be used in both nuclear and medical applications. Camgöz et al. conducted an experimental investigation to test the gamma-shielding properties of natural stones [26]. Gümrükçüoğlu et al. determined the gamma-ray attenuation properties of spilitic basalt, andesite, dolomite, limestone, and syenite rocks [27]. The study revealed that andesite rocks are more effective for radiation shielding compared to the other rocks studied. This research likely explores how rocks may contribute to or impede the penetration of photons, with potential implications for radiation protection and related fields.

The current study aims to identify more effective gamma-ray-shielding natural materials for reliable use in radiation research including basalt, bentonite, sand, and claystone. It studied, for different samples, the mass and linear attenuation coefficients of gamma rays at energy levels of 662, 1173, and 1332 keV. Additionally, parameters like the tenth-value layer, half-value layer, and mean free path were calculated based on the experimental data. The calculated (μ/ρ) values were contrasted with theoretical outcomes acquired using the Win XCOM program. Finally, assess environmental radiation hazards by determining the concentrations of naturally occurring radioactive elements, including ^238^U, ^226^Ra, ^232^Th, and ^40^K. This involved analyzing samples through various techniques such as gamma-ray spectroscopy and subsequently calculating hazard indices such as internal hazard index, external hazard index, and radium equivalent activity. The results were then evaluated against established safety standards to gauge environmental radiation risks.

## 2. Materials and Methods

Six different rock samples were collected using the channel sampling method from various localities in Egypt. The first group consisted of four samples gathered from southwestern Sinai, east Abu Zeneima, Egypt. These included Basalt-FA from the Farsh El Azraq locality, bentonitic shale, bentonitic claystone, and sandstone. The second group, comprising Basalt-AZ and claystone, was obtained from the Abouzabal region and the EL Qattamiya asphaltic road, respectively.

For measuring the attenuation parameter experimentally, each sample was divided into four cylindrical pellets of varying thickness. Using a well-calibrated gamma-ray spectrometer consisting of a 3 × 3″NaI(Tl) scintillation detector, the attenuation coefficient of each sample was measured for gamma rays of 662 keV energy of ^137^Cs and 1173 and 1332 keV energies of ^60^Co. A lead collimator was used to make sure that the NaI detector absorbed a narrow beam of gamma rays after it passed through the test column to precisely account for the absorption of gamma radiation in the samples under investigation. All samples’ chemical compositions were determined using an X-ray fluorescence analysis (X-supreme 8000) apparatus. The XRF technique used energy-dispersive X-ray fluorescence (EDXRF model NEX CG). Dried powdered samples (<200 mesh) were prepared to minimize the mineralogical effect and reduce extraneous X-ray scattering and then mixed and compressed with boric acid.

Utilizing a Malvern Panalytical Empyrean X-ray diffractometer with Cu Kα1 radiation (λ = 1.5418 Å) and a tube voltage and current of 40 kV and 30 mA, respectively, XRD patterns were obtained. The range of diffraction patterns recorded was 5 to 75° (2θ), and they were compared with pre-existing patterns found in powder diffraction files (PDFs). Bragg’s law was applied to determine the separation between the silicate layers. Particle size and lattice strain resulting from sample dislocation or size reduction can be assessed using XRD analysis. For this, Williamson–Hall analysis was utilized [28], beginning at a position of 4.9950 [°2Th], concluding at 75.0150 [°2Th.], with a step size of 0.0200 [°2Th.], a scan step time of 0.6000 s, employing continuous scanning mode, with a total duration of approximately 35 min.

A 76 × 76 mm Bicron scintillation detector and NaI (Tl) crystal made up the gamma-ray spectrometry system. The measurement of the radionuclides was based on selecting four energy regions of interest (ROIs) from the standard samples NMA-U, IAEA-Th, IAEA-Ra, and IAEA-K obtained from the International Atomic Energy Agency [29]. These ROIs represented ^234^Th, ^212^Pb, ^214^Pb, and ^40^K for U, Th, Ra, and K, respectively, and four standard natural rock samples, NMA-U, IAEA-Th, IAEA-Ra, and IAEA-K, used to evaluate the efficiency of studied rock (bentonitic shale, sandstone, and Basalt-AZ) samples for attenuation and their abilities to reduce radiological hazard.

### 2.1. Shielding Parameters

The three primary methods used to reduce the risks of radiation from the outside are distance, time, and shielding material. Sophisticated techniques for radiation protection must be optimized to be effective. Time, the duration of exposure, significantly influences the radiation dose an individual accumulates. Distance, the distance from the radiation source, is a critical factor in reducing exposure. The amount of radiation an individual receives decreases as they move farther away from the radiation source. The relationship between intensity and distance follows the inverse square law for point sources of x and gamma radiation (I_1_d_1_^2^ = I_2_d_2_^2^, where intensity I_1_ is measured at distance d_1_ and intensity I_2_ is measured at distance d_2_).

Shielding utilizing protective barriers or shielding materials can effectively reduce radiation exposure. The choice of shielding material depends on the type and energy of the radiation. The shielding material acts as a barrier that absorbs or deflects radiation, preventing it from reaching individuals and reducing their exposure. In summary, optimizing time, maintaining a safe distance from the radiation source, and implementing appropriate shielding materials are fundamental strategies for effective radiation protection.

The linear attenuation coefficient (μ) is an important parameter for evaluating the interaction between gamma radiation and matter. This coefficient can be determined using the Beer–Lambert law [25]. This law provides a method for calculating the attenuation of radiation as it passes through a material and is expressed as follows:

(1) $$\mu =\frac{1}{x}\ln\;(\frac{I_{0}}{I})$$

The calculation of the linear attenuation coefficient (μ) involves comparing the intensity of incident gamma-ray photons $I_{0}$ with the transmitted gamma-ray photons I through an absorber of thickness x. The values of I and $I_{0}$ are determined by measuring the peak count rate in the absence and presence of the studied rock samples, respectively. The graphing of the correlation between the thickness (x, cm) of the fabricated concrete and the ln (Io/I) yielded the μ values [30,31].

Additionally, the coefficient of mass attenuation ($\mu_{m}$) is computed to assess the shielding capability of the concerned materials against gamma rays, independent of material density. This is achieved by dividing the experimentally determined linear attenuation coefficient (μ) for a specific material by its density (ρ). The theoretical calculation of μ/ρ can also be performed using Equation (2) [32]:

(2) $$\frac{\mu }{\rho }=\;\sum_{i}w_{i}\;{\left(\frac{\mu }{\rho }\right)}_{i}$$

where ${\left(\frac{\mu }{\rho }\right)}_{i}$ is the coefficient of mass attenuation for the $i_{th}$ constituent element and $w_{i}\;$ is the weight fraction of that element in the sample. Equation (3) is used to calculate the standard deviation between experimental and theoretical mass attenuation coefficient values that were calculated using the XCOM program:

(3) $$Deviation\;(\%)\;=\frac{\mu_{m}(theoretical)-\mu_{m}(experimental)}{\mu_{m}(theoretical)}\;\times \;100$$

The half-value layer (HVL), a crucial parameter for assessing radiation protection materials, represents the thickness required to reduce incident radiation to 50% of its initial value. The HVL is evaluated using Equation (4) [33].

(4) $$HVL=\frac{\ln\left(2\right)}{µ}$$

The tenth-value layer (TVL) is a significant parameter indicating the thickness of a shielding material required to reduce gamma radiation to one-tenth of its initial intensity. The estimation of TVL is typically performed using Equation (5) [33].

(5) $$TVL=\frac{\ln\left(10\right)}{µ}$$

The mean free path (MFP) is the average distance a photon takes between two successive interactions. Equation (6) describes this path.

(6) $$MFP=\frac{1}{\mu }$$

### 2.2. Environmental Hazards

#### 2.2.1. Absorbed Dose and Annual Effective Dose

The transformation of the radionuclide activity concentrations of U238, Th232, and K40 in the obtained samples into doses is necessary to calculate the absorbed and annual effective dose. This process involves multiplying each radionuclide by factors of 0.462, 0.604, and 0.0417 (nGy h^−1^ per Bq kg^−1^), respectively. Consequently, the total absorbed gamma dose (D) can be represented as follows (UNSCEAR, 2000) [34]:

(7) $$D\left(nGyh^{-1}\right)=0.462C_{U}+0.604C_{Th}+0.0417C_{K}$$

Equations that account for both indoor and outdoor occupancy, along with the conversion factor that is provided, can be used to calculate an individual’s yearly conjectural average effective dose equivalent. The following equations are used to calculate the annual effective doses (AEDR):

(8) $$\mathrm{Indoor}\;(\mathrm{mSv})=D\;nGyh^{-1}\times \;8760\;h\;\times \;0.8\;\times \;0.7\;\mathrm{Sv}\;{\mathrm{Gy}}^{-1}$$

(9) $$\mathrm{Outdoor}\;(\mathrm{mSv})=D\;nGyh^{-1}\times 8760\;h\times 0.8\times 0.7\;\mathrm{Sv}\;{\mathrm{Gy}}^{-1}$$

where *D* is the total absorbed gamma dose obtained from the previous calculation, 8760 h represents the number of hours in a year, 0.80 and 0.20 are the proportions of time spent indoors and outdoors, respectively, and 0.7 Sv Gy^−1^ is the conversion factor.

#### 2.2.2. Computing of Radium Equivalent

The radium equivalent activity (${Ra}_{eq}$) is a radiation hazard indicator that represents the equivalent activity of natural radionuclides and their contributions to the gamma-ray dose rate. It is calculated with the following equation [35]:

(10) $${Ra}_{eq}=\;C_{U}+\;1.43C_{Th}+\;0.077C_{K}$$

where *C*_*U*_ is the ^238^*U* concentration of activity, $C_{Th}$ is the ^232^*Th* concentration of activity, and $C_{K}\;$ is the ^40^*K* concentration of activity (Bq kg^−1^). The coefficients (1.43 and 0.077) are used to weight the contributions of ^232^*Th* and ^40^*K* relative to ^238^*U* in terms of gamma-ray dose rate.

#### 2.2.3. Identification of Radiological Hazards

Hazard indices are frequently used to indicate the radiological hazards associated with radionuclides such as Ra (226U)238, Th232, and K40. The hazard indices aid in evaluating the possible radiological dangers associated with internal ($H_{in}$), and external ($H_{ex}$) exposure along with the gamma representative index (*Iγ*). The following formulas are used to calculate the indices [36,37,38,39]:

(11) $$H_{ex}=\frac{C_{u}}{370}+\;\frac{C_{Th}}{259}+\;\frac{C_{K}}{4810}$$

(12) $$H_{in}=\frac{C_{u}}{185}+\frac{C_{Th}}{259}+\frac{C_{K}}{4810}$$

(13) $$I_{\gamma }=\frac{C_{u}}{300}+\frac{C_{Th}}{200}+\frac{C_{K}}{300}$$

where $C_{Ra}$, $C_{Th}$, and $C_{K}\;$ are the activity concentrations of Ra (226U)238, Th232, and K40 in Bq kg ^−1^, respectively. To restrict external gamma radiation doses below 1.5 mGy per year, the external hazard index $H_{ex}$ must not exceed 1. This safety measure is essential for keeping radiation exposure levels within acceptable limits, protecting human health, and reducing any potential negative effects from exposure to external gamma radiation.

A metric called the annual gonad dose equivalent (AGDE) measures the genetic effect of the yearly dose equivalent that a population’s reproductive organs receive. The following equation is commonly used to calculate AGDE [37,38,39]:

(14) $$AGDE\left(\frac{mSv}{y}\right)=3.09C_{u}+4.18C_{Th}+0.314C_{K}$$

## 3. Results and Discussion

A few naturally occurring rocks, like Basalt-AZ, Basalt-FA, bentonitic shale, bentonitic claystone, sandstone, and claystone have been tested for their nuclear shielding efficiency. According to an X-ray fluorescence analysis (XRF) test, the weight fraction of elements in these rocks is provided in Table 1. With this elemental information, the coefficient of mass attenuation can be calculated using the Win XCom program, enabling a more comprehensive understanding of the materials’ interaction with gamma radiation. This approach enhances the assessment of the nuclear shielding efficiency of the studied rocks.

**Figure 1:**
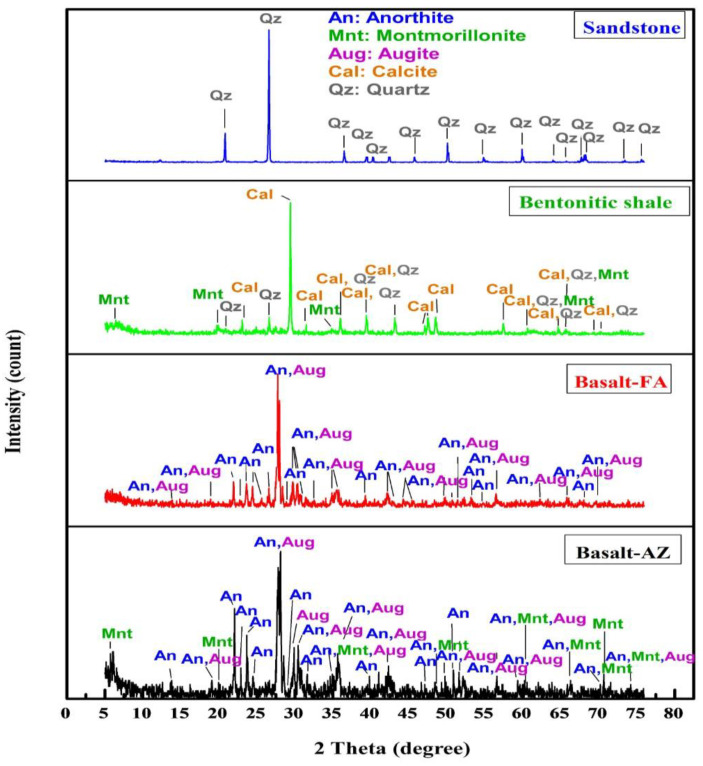
The structure properties of different natural materials (sandstone, bentonitic shale, Basalt-FA, and Basalt-AZ) as determined by X-ray diffraction.

The mineral compositions of different natural materials (Basalt-AZ, Basalt-FA, bentonitic shale, and sandstone) can be identified by X-ray diffraction as shown in Figure 1. The X-ray diffraction (XRD) patterns for both basalt samples from different regions reveal the presence of two major crystalline phases: anorthite and augite [40]. These phases are identified based on the Joint Committee of Powder Diffraction Standard (JCPDS) card numbers 41-1486 and 24-0201, respectively. In the case of a basalt sample obtained from the Abouzabal region, an additional phase is observed. This phase is identified as montmorillonite, and its presence is confirmed by the JCPDS card number 3-0015.

The X-ray diffraction (XRD) analysis of the bentonitic shale sample reveals the presence of three different crystalline phases: calcite, identified with the Joint Committee of Powder Diffraction Standard (JCPDS) card number 072-1937; quartz, identified with the JCPDS card number 85-0794; montmorillonite, identified with the JCPDS card number 3-0015.

On the other hand, the XRD analysis of the sandstone sample indicates the presence of only one crystalline phase, quartz, identified with the JCPDS card number 85-0794. These findings provide insights into the mineral composition of the bentonite and sandstone samples, highlighting the specific crystalline phases present in each sample. Table 2 illustrates the different types of samples used in this study and their specific mass density.

The determination of particle size for the four natural samples involves using XRD analysis and applying Williamson–Hall size analysis. This method considers lattice strain arising from various sources such as lattice dislocation, crystal defects, and grain boundaries. One significant contributor to lattice strain is size reduction (grinding), which induces many dislocations in the crystal structure. The Williamson–Hall equation used for the calculation is as follows:

(15) $$\beta_{hkl}\cos\theta =\;\frac{K\lambda }{D}+4\epsilon \;sin\theta$$

where $\beta_{hkl}$ is the full width at half maximum (FWHM), θ is the Bragg angle, K is the shape factor, λ is the wavelength, D is the measured particle size, and ϵ is the strain resulting from crystal defects [41,42,43].

**Figure 2:**
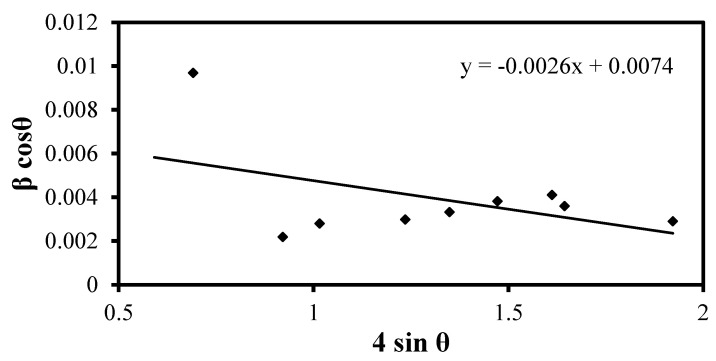
W-H size analysis for bentonite clay.

**Figure 3:**
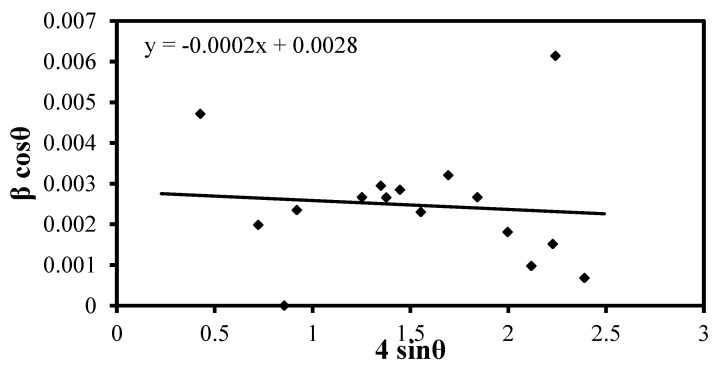
W-H size analysis of sand sample.

The provided information details the particle size determination for bentonitic shale and sandstone using Williamson–Hall size analysis based on Figure 2 and Figure 3. The particle size of bentonitic shale is 188 nm, determined from the intercept with the y-axis (0.0074), and the strain value is determined from the slope of the Williamson–Hall equation to be 0.0026. The particle size of the sandstone sample is 495 nm, and the strain value is 0.0028, as determined from Figure 3.

**Figure 4:**
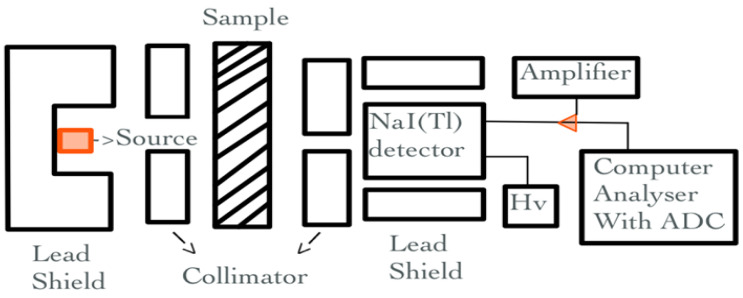
Schematic diagram of the experimental set-up.

The attenuation coefficients of each sample were measured for gamma rays of 662 keV energy of ^137^Cs and 1173 and 1332 keV energies of ^60^Co sources, using a gamma-ray spectrometer equipped with a 3″ × 3″ NaI(Tl) scintillation detector. The detector was shielded from induced X-rays by a cylindrical copper barrier and a chamber of lead bricks against ambient radiation. The detector was linked to Nuclear Enterprises’ main shaping amplifier as well as a high-voltage power supply with an HV digital display. The detector was additionally linked to a Nuclease PCA-8000 computer-based 8192 multichannel analyzer with color graphical spectra display and advanced technical operation features. The narrow beam geometry with a lead collimator was utilized for the absorption of gamma radiation in the materials under investigation, as shown in Figure 4.

The study involves the calculation of key parameters such as the linear attenuation coefficient, mass attenuation coefficient, half-value layer, tenth-value layer, and mean free path for six types of shielding materials, namely basalt AZ, basalt FA, bentonitic shale, bentonitic claystone, sandstone, and claystone, with ^137^Cs and ^60^Co radioactive sources. Table 3 presents the experimental values of the linear attenuation coefficient for these materials, obtained by measuring the intensities of gamma rays passing through the absorbers. The results indicate that basalt, a sample obtained from the Abouzabal region, has the highest linear attenuation coefficient (0.225 cm^−1^ at 661.66 keV, 0.1675 cm^−1^ at 1173.47 keV and 0.1604 cm^−1^ at 1332.51 keV), while bentonitic claystone has the lowest (0.136 cm^−1^ at 661.66 keV, 0.1051 cm^−1^ at 1173.47 keV and 0.1014 cm^−1^ at 1332.51 keV). This observation is attributed to the presence of montmorillonite, Figure 1, which is a type of clay mineral whose composition includes layers of aluminum and magnesium octahedra sandwiched between layers of silica tetrahedra, making it more effective for gamma-ray attenuation.

Furthermore, this study notes that the linear attenuation coefficient values decrease with increasing incident energy. This dependency indicates that the linear attenuation coefficient is influenced by the energy of the incident gamma radiation.

The mass attenuation coefficient is another crucial parameter measured in this study to understand gamma-ray interactions with different materials. The experimental values of the mass attenuation coefficient for the six-shielding materials basalt AZ, basalt FA, bentonitic shale, bentonitic claystone, sandstone, and claystone are presented in Table 3, and the calculations were performed using Equation (4). From the results, it is observed that bentonitic shale consistently exhibits a higher experimental mass attenuation coefficient for 1173.47 and 1332.51(keV) (µ_mexp_) gamma sources compared to the other shielding materials. This is attributed to the specific composition of bentonitic shale, including minerals such as montmorillonite, feldspar, quartz, illite, smectite, kaolinite, chlorite, and potentially others [44]. Furthermore, this study notes a good agreement between the experimental and theoretical values, using the XCOM program, of the mass attenuation coefficient. The mass attenuation coefficients were determined using experimental linear attenuation values for the studied samples at various energies compared with other values globally presented in Table 4.

**Figure 5:**
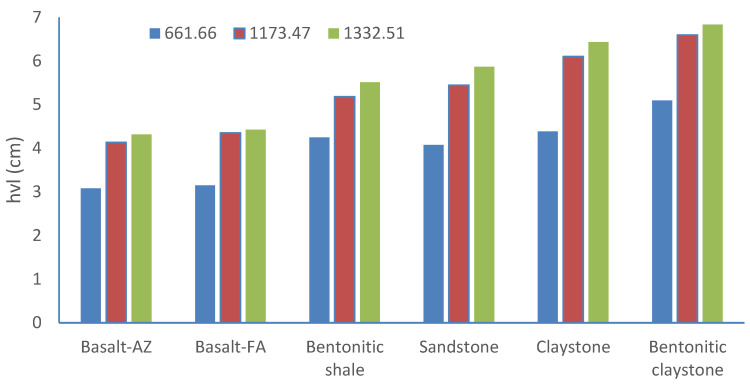
HVL for different natural rock samples at different energies.

The half-value layer (HVL), tenth-value layer (TVL), and mean free path (MFP) are critical parameters in radiation-shielding design. They determine the effectiveness of materials in attenuating radiation. Table 5 provides the HVL, TVL, and MFP values for different shielding materials at 661.66, 1173.47, and 1332.51 keV respectively. The analysis reveals that the bentonitic claystone sample exhibits the highest values for half-value layer (HVL), tenth-value layer (TVL), and mean free path (MFP), indicating lower shielding performance. Conversely, basalt, a sample obtained from the Abouzabal region, demonstrates the lowest values for these parameters, suggesting superior shielding capabilities, as shown in Figure 5.

The activity concentrations of ^238^U, ^232^Th, ^226^Ra, and ^40^K radionuclide were determined using ROIs corresponding to the daughter nuclides ^234^Th, ^212^Pb, ^214^Pb, and ^40^K, respectively. These measurements were conducted using a gamma-ray spectrometer equipped with a NaI(Tl) scintillation detector at specific energies: 92.60 keV, 238.6 keV, 352.0 keV, and 1460.0 keV. Furthermore, the mass attenuation coefficient (${µ}_{m}$) for different types of shielding materials, including basalt, bentonitic shale, and sandstone, was calculated using the XCOM program at these specific energies. According to the results presented in Table 6, bentonitic shale consistently exhibits a higher mass attenuation coefficient across all gamma sources compared to the other shielding materials.

**Figure 6:**
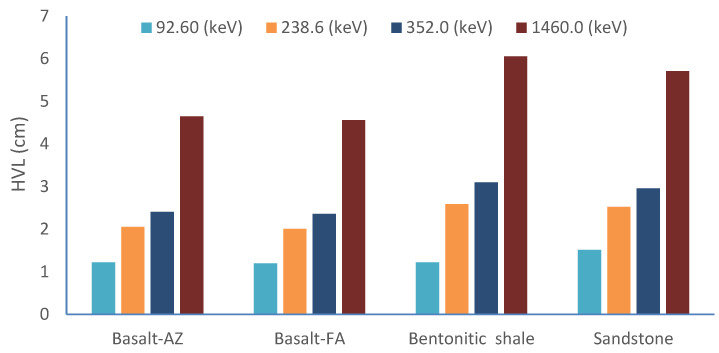
The HVL calculated from theoretical mass attenuation (µ_m_) by XCOM program at energies.

The analysis of Table 6 and Figure 6 shows that bentonitic shale has the highest values of the half-value layer (HVL) for all studied energies, except for the energy at 92.60 keV. This indicates its lower shielding performance. In contrast, Basalt-FA and Basalt-AZ, from southwestern Sinai and Abouzabal, exhibit the lowest HVL values, suggesting superior shielding capabilities. Table 7 demonstrates that the sandstone rock samples consistently show a half-value layer (HVL) lower than bentonitic shale for all studied energies, except at 92.60 keV, corresponding to the calculation of Uranium-238 (^238^U). At this specific energy level, the sandstone sample exhibits a higher HVL value compared to bentonitic shale.

Table 7 presents the results obtained from utilizing standard radionuclide samples (NMA-U, IAEA-Th, IAEA-Ra, and IAEA-K) provided by the International Atomic Energy Agency (IAEA, Matolin, 1991) to evaluate the attenuation efficiency (%) of the studied rock samples (bentonitic shale, sandstone, and Basalt-AZ) at two thicknesses (1.5 and 2 cm) [29].

At 1.5 cm between the counting detector and the measured standard samples without rock samples, the activity attenuation percentages for ^238^U, ^232^Th, ^226^Ra, and ^40^K were 25.3%, 32.9%, 33.7%, and 34.9%, respectively, for the standards NMA-U, IAEA-Th, IAEA-Ra, and IAEA-K. At 2 cm, the activity attenuation percentages for ^238^U, ^232^Th, ^226^Ra, and ^40^K were 33.73%, 43.87%, 44.93%, and 46.53%, respectively, for the same standards.

The results from Table 7 demonstrate that Basalt-AZ, sourced from the Abouzabal region, shows the highest attenuation percentages for all standard samples. At a thickness of 1.5 cm, Basalt-AZ achieves attenuation percentages of 68.58% for NMA-U, 56.51% for IAEA-Th, 58.42% for IAEA-Ra, and 49.99% for IAEA-K. When the thickness of Basalt-AZ is increased from 1.5 cm to 2 cm, the attenuation percentage rises by approximately 11%, reaching values of 77.12%, 67.2%, 67.65%, and 59.8% for NMA-U, IAEA-Th, IAEA-Ra, and IAEA-K, respectively.

**Figure 7:**
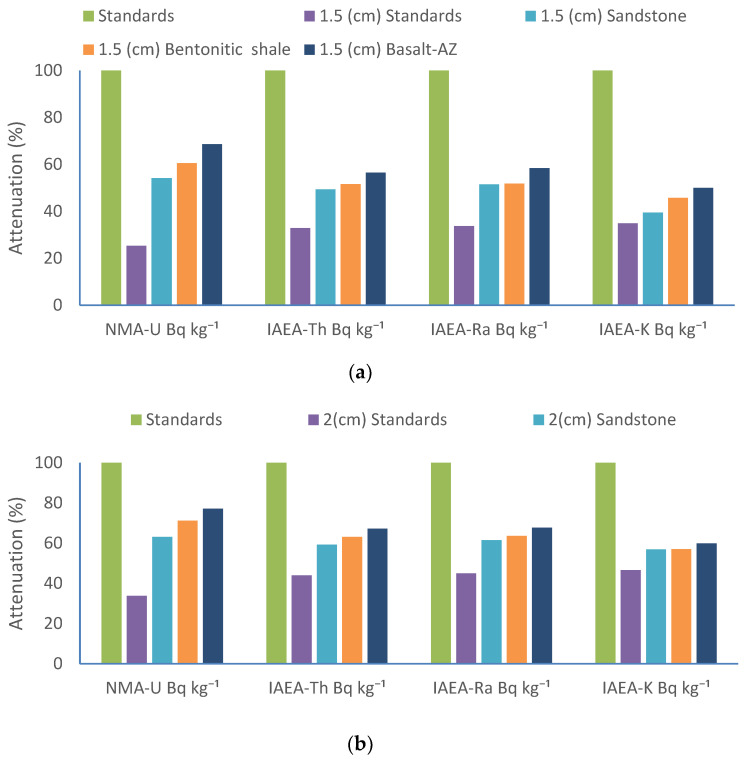
Attenuation (%) for the standard radionuclides and the studied rock (bentonitic shale, sandstone, and Basalt-AZ) samples at (**a**) 1.5 cm and (**b**) 2 cm from the NaI (Tl) detector.

At a thickness of 1.5 cm, Basalt-AZ demonstrated the highest attenuation percentages (%), followed closely by bentonitic shale and then sandstone. Specifically, bentonitic shale displayed percentages of 60.55%, 51.57%, 51.85%, and 45.72%, while sandstone showed the lowest percentages of 54.17%, 49.39%, 51.51%, and 39.44%. The attenuation percentages were analyzed for the investigated samples in Figure 7a,b.

At a thickness of 2 cm, the trend persisted, with Basalt-AZ consistently exhibiting the highest attenuation percentages, followed by bentonitic shale and then sandstone. These results indicate that Basalt-AZ showcases superior attenuation capabilities through the different standard samples, with its efficiency in attenuation further increasing with thicker material thicknesses, as shown in Figure 7b.

It is important to highlight that the effectiveness of these rocks in attenuating radiation led to a reduction in activity in standard samples by over 50%, even at thicknesses below the theoretically calculated half-value layer (HVL). This reduction is due to both the attenuation through the rocks and the distance between the measured standard samples and the NaI (Tl) scintillation detector.

Moreover, while the half-value layer (HVL) concept is primarily designed for narrow radiation beams, wide radiation beams experience significant propagation. This propagation diminishes the efficacy of the HVL system in attenuating the rays because there are fewer opportunities for interaction between the radiation and the attenuating material.

**Figure 8:**
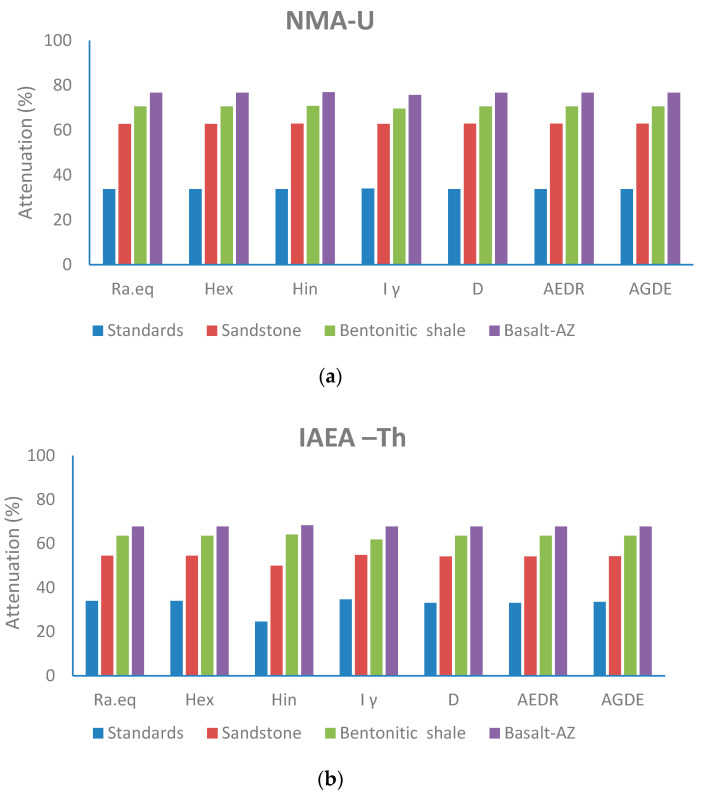

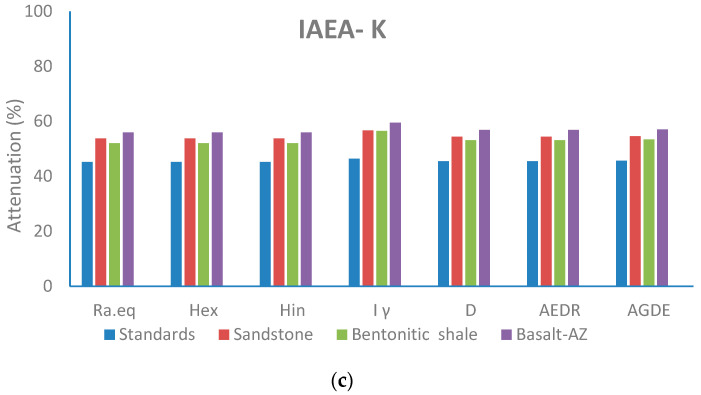
The attenuation efficiency (%) of radiological hazard at 2 cm for bentonitic shale, sandstone, basalt (Basalt-AZ), and standard radionuclide samples: (**a**) NMA-U, (**b**) IAEA-Th, and (**c**) IAEA-K.

Table 8 and Table 9 present a comprehensive assessment of radiological hazard effects based on the activity concentration of primordial radioactive elements ^238^U (^226^Ra), ^232^Th, and ^40^K. Various radiological hazards were calculated using specified parameters to evaluate their impact on surrounding living biota. The efficiency of the studied rock (bentonitic shale, sandstone, and Basalt-AZ) samples in attenuating the radiological hazard (%) was evaluated at two thicknesses (1.5 and 2 cm). The radiological hazards considered include radium equivalent (Ra_eq_), external (H_ex_) and internal exposure (H_in_), absorbed dose (D) in nG/h, annual effective dose (AEDE) in mSv/y, and the annual gonad dose equivalent (AGDE) in mSv/y. Figure 8 illustrates the attenuation (%) for the standard radionuclide samples NMA-U, IAEA-Th, and IAEA-K obtained from the International Atomic Energy Agency [29]. These values are utilized to assess the efficiency of the studied rocks (bentonitic shale, sandstone, and Basalt-AZ) in attenuating the radiological hazard (%) at 2 cm thickness.

For the NMA-U standard, the data demonstrate the highest attenuation (%) values for all radiological hazards. At a 2 cm thickness, the attenuation (%) values for Basalt-AZ, bentonitic shale, and sandstone are 68.4%, 60.3%, and 54.1%, respectively. In contrast, the IAEA-Th standard shows lower attenuation (%) values for all radiological hazards, ranging from 67.7% to 68.3% at a 2 cm thickness for Basalt-AZ and from 61.8% to 64.1% for bentonitic shale. Meanwhile, sandstone exhibits the lowest attenuation (%) values, ranging from 50% to 54.5% at a 2 cm thickness. Additionally, for the IAEA-K standard, the attenuation (%) values for all radiological hazards range from 55.9% to 59.5% at a 2 cm thickness for Basalt-AZ and from 52% to 56.5% at the same thickness for bentonitic shale.

The analysis of radiation hazards indicates that sandstone consistently exhibits lower attenuation ratios for all parameters compared to basalt and bentonitic shale in NMA-U and IAEA-Th, as shown in Figure 8. This aligns with previous findings, except for the percentage attenuation for the IAEA-K standard. Notably, the sandstone attenuation ratio of ^40^K is very close to that of basalt and bentonite. This observation can be attributed to the absence of potassium in sandstone, whereas both basalt and bentonitic shale contain a small percentage of K_2_O in their composition (0.55% and 1.12%, respectively). The presence of potassium in basalt and bentonitic shale negatively impacts the attenuation value, reducing their efficiency in attenuating ^40^K activity.

## 4. Conclusions

Gamma radiation can be attenuated using a wide range of materials. Understanding how gamma rays are attenuated due to physical photon–matter interactions aids in selecting appropriate shielding materials for specific applications. The development of this understanding while considering physical, chemical, and economic limits results in more effective resource utilization for developing suitable shielding. Measurements of the mass attenuation coefficient (μ_m_) and related parameters such as half-value layer (HVL), tenth-value layer (TVL), and mean free path (MFP) have been conducted using NaI(Tl) detectors. Basalt, due to its wide availability and affordable price, emerges as a viable option for shielding. Moreover, natural bentonitic shale and sandstone, found naturally on a nanometer scale, exhibit efficient attenuation properties against gamma-ray radiation. The results indicate that as thickness increases, linear and mass attenuation coefficients also increase. Basalt samples demonstrate superior shielding parameters compared to other rock samples, attributed to their higher densities. Experimental values align well with theoretical values estimated using the XCOM program. Based on HVL, TVL, and MFP values, natural rock materials hold promise for radiation protection applications, shielding individuals from the damaging effects of gamma radiation. Based on this study, basalt, due to its worldwide abundance and the toxicity of conventional lead shielding, is seen as a feasible alternative for radiation protection. Basalt can effectively shield against harmful gamma radiation and is suitable for use in both medical and industrial applications. However, it is recommended to use basalt fibers infused with a boron oxide additive as a dispersed concrete reinforcement to enhance the material’s radiation-shielding properties.

## Figures and Tables

**Table 1:** The chemical composition of different natural rocks using XRF.

Rock Type	Oxides (%)													
	SiO_2_	Al_2_O_3_	P_2_O_5_	TiO_2_	Fe_2_O_3_	CaO	MgO	MnO	Na_2_O	K_2_O	L.O.I (550)	L.O.I (1000)	Total L.O.I	Total
Basalt-AZ (Abou Zabal)	52.3	17.61	0.11	1.58	9.41	6.5	6.32	0.127	3.96	0.55	1.39	0.14	1.53	100.0
Basalt-FA (Farsh El Azraq)	50.53	17.72	0.171	2.11	11.3	8.32	3.6	0.176	4.5	0.63	0.81	0.08	0.89	99.9
Bentonitic shale	23.47	14.88	0.146	0.413	4.19	23.7	2.98	0.467	2	1.12	8.73	17.69	26.69	99.8
Sandstone	82.04	2.55	0.622	0.07	6.48	2.92	0.81	0.69	0.03	0.143	0.76	1	1.76	98.1
Claystone	47.33	23.21	0.05	0.86	4.67	1.12	0.86	-	1.65	1.63	14.23	4.40	18.63	100.0
Bentonitic claystone	61.54	14.54	0.17	0.53	1.68	0.65	0.25	-	0.8	6.92	8.02	6.32	14.34	101.4

**Table 2:** The different types of the studied natural rocks.

Sample Name	Density g/cm^3^	Shape
Basalt-AZ (Abou Zabal)	2.86	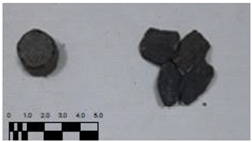
Basalt-FA (Farsh El Azraq)	2.923	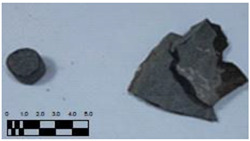
Bentonitic shale	2.197	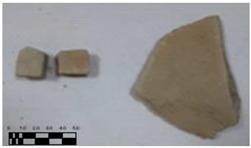
Sandstone	2.322	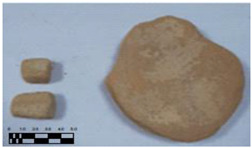
Claystone	1.987	^ 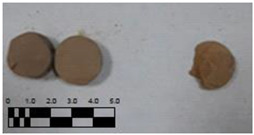 ^
Bentonitic claystone	1.809	^ 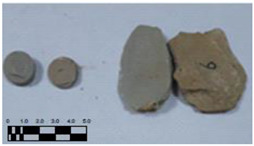 ^

**Table 3:** The deviation between theoretical and experimental mass attenuation coefficients at energies 661.660 (keV), 1173.47 (keV), and 1332.51 (keV).

Sample Type	ρ (g/cm^3^)	661.660 (keV)					1173.47 (keV)				1332.51 (keV)		
		µ	µ_m_ (exp)	µ_m_ (theo)	Devi. (%)	µ	µ_m_ (exp)	µ_m_ (theo)	Devi. (%)	µ	µ_m_ (exp)	µ_m_ (theo)	Devi. (%)
Basalt-AZ	2.86	0.225	0.0786	0.0767	−2.48	0.1675	0.0586	0.0583	−0.51	0.1604	0.0561	0.0547	−2.56
Basalt-FA	2.923	0.220	0.0754	0.0766	1.57	0.1593	0.0545	0.0582	6.36	0.1566	0.0536	0.0545	1.65
Bentonitic shale	2.197	0.163	0.0742	0.0685	−8.32	0.1337	0.0608	0.0582	−4.47	0.1257	0.0572	0.0546	−4.76
Sandstone	2.322	0.170	0.0730	0.0769	5.07	0.1272	0.0548	0.0585	6.32	0.1181	0.0509	0.0548	7.12
Claystone	1.987	0.158	0.0796	0.0772	−3.11	0.1135	0.0571	0.0588	2.89	0.1077	0.0542	0.0552	1.81
Bentonitic claystone	1.809	0.136	0.0752	0.0772	2.59	0.1051	0.0581	0.0588	1.19	0.1014	0.0561	0.0552	−1.63

**Table 4:** Comparing the mass attenuation coefficients (cm^2^.g^−1^) of the tested samples at the investigated gamma energies to those of prior research conducted globally.

Sample Type	Energy (keV)			Country	Reference
	661.66	1173.47	1332.51		
Basalt-AZ	0.0786	0.0586	0.0561	Egypt	Present work
Basalt-FA	0.0754	0.0545	0.0536		
Bentonitic shale	0.0742	0.0608	0.0572		
Sandstone	0.0730	0.0548	0.0509		
Claystone	0.0796	0.0571	0.0542		
Bentonitic claystone	0.0752	0.0581	0.0561		
Bentonite Clay	0.0780	0.0570	0.0540	Egypt	Elsafi, M. et al. [45]
bentonite–gypsum	0.0787	0.0586	0.0538	Egypt	Mahmoud I. Abbas et al. [46]
Olivine basalt	0.076 0	0.0590	0.0550	India	Obaid et al. [14]
Bentonite/PVA	0.0760	0.0590	0.0560	Egypt	Fawzy H. S. et al. [47]
Natural bentonite	0.0760	0.0590	0.0543	Egypt	Ibrahim, Z. H. et al. [48]
High consistency concrete	Range (0.08–0.078)	Range (0.053–0.057)	Range (0.053–0.054)	Turkey	Gökçe et al. [49]
Polyboron	0.0870	0.0660	0.0620	Bangladesh	Biswas et al. [50]
Ordinary concrete	0.0780	0.0590	0.0550		
Olivine Basalt	0.0766	0.0581	0.0544	Turkey	Al-Buriahi, M. S. et al. [19]
Sandstone	0.0772	0.0587	0.0550		
Basalt	0.0544	0.0538	0.0380	Turkey	Gümrükçüoğlu, N. et al. [27]

**Table 5:** HVL, TVL and MFP for different natural rock samples at different energies.

Sample Type	HVL (cm)			TVL (cm)			MFP (cm)		
	661.660	1173.47 (keV)	1332.51	661.660	1173.47 (keV)	1332.51	661.660	1173.47 (keV)	1332.51
Basalt-AZ	3.080	4.137	4.320	10.222	13.731	14.339	4.444	5.970	6.234
Basalt-FA	3.150	4.350	4.425	10.455	14.438	14.687	4.545	6.277	6.386
Bentonitic shale	4.252	5.183	5.513	14.110	17.203	18.298	6.135	7.479	7.955
Sandstone	4.076	5.448	5.868	13.529	18.082	19.475	5.882	7.862	8.467
Claystone	4.386	6.106	6.435	14.557	20.264	21.356	6.329	8.8106	9.285
Bentonitic claystone	5.096	6.594	6.834	16.912	21.884	22.682	7.353	9.515	9.861

**Table 6:** The theoretical mass attenuation (µ_m_) by XCOM program and HVL at energies 92.60 (keV), 238.6 (keV), 352.0 (keV), and 1460.0 (keV).

Sample Type	92.60 (keV) (^238^U)			238.6 (keV) (^232^Th)			352.0 (keV) (^226^Ra)			1460.0 (keV) (^40^K)		
	(µ_m_)	µ	HVL	(µ_m_)	µ	HVL	(µ_m_)	µ	HVL	(µ_m_)	µ	HVL
Basalt-AZ	0.198	0.567	1.223	0.118	0.337	2.054	0.1006	0.288	2.409	0.0522	0.149	4.646
Basalt-FA	0.198	0.579	1.198	0.118	0.345	2.011	0.1005	0.294	2.360	0.0520	0.152	4.558
Bentonitic shale	0.258	0.567	1.223	0.122	0.267	2.592	0.1018	0.224	3.099	0.0521	0.115	6.052
Sandstone	0.197	0.457	1.515	0.118	0.274	2.525	0.1009	0.234	2.959	0.0523	0.121	5.708

**Table 7:** The attenuation efficiency (%) of the studied rock samples (bentonitic shale, sandstone, and Basalt-AZ) at 1.5 and 2 cm thicknesses using standard radionuclide samples MA-U, IAEA-Th, IAEA-Ra, and IAEA-K.

Standard Thickness	Sample Type	NMA-U Bq kg^−1^	IAEA-Th Bq kg^−1^	IAEA-Ra Bq kg^−1^	IAEA-K Bq kg^−1^	NMA-U Attenuation (%)	IAEA-Th Attenuation (%)	IAEA-Ra Attenuation (%)	IAEA-K Attenuation (%)
		12,691.03	3232.00	4351.20	14,022.40				
1.5 (cm)	Standards	9480.20	2168.67	2884.85	9128.58	25.30	32.90	33.70	34.90
	Sandstone	5816.84	1635.80	2110.11	8482.30	54.17	49.39	51.51	39.44
	Bentonitic shale	5007.12	1565.10	2094.57	7605.90	60.55	51.57	51.85	45.72
	Basalt-AZ	3987.84	1405.52	1809.30	7011.20	68.58	56.51	58.42	49.99
2 (cm)	Standards	8409.92	1814.23	2396.07	7497.31	33.73	43.87	44.93	46.53
	Sandstone	4693.40	1319.46	1678.32	6040.90	63.02	59.18	61.42	56.84
	Bentonitic shale	3670.40	1194.63	1585.08	6040.90	71.08	63.04	63.57	57.02
	Basalt-AZ	2904.08	1060.10	1407.48	5634.00	77.12	67.20	67.65	59.80

**Table 8:** The attenuation efficiency (%) of radiological hazard for bentonitic shale, sandstone, and basalt (Basalt-AZ) at 1.5 cm thickness and standard radionuclide samples NMA-U, IAEA-Th, and IAEA-K.

1.5 (cm)														
	Ra.eq	Hex	Hin	I γ	D nG/h	AEDE mSv/y	AGDE mSv/y	Ra.eq	Hex	Hin	I γ	D nG/h	AEDR mSv/y	AGDE mSv/y
NMA-U	12,705.1	34.3	68.6	42.5	5869.8	7.2	39,262.0			Attenuation (%)				
Standards	9499.3	25.7	51.3	31.8	4388.3	5.4	29,353.1	25.2	25.2	25.3	25.3	25.2	25.2	25.2
Sandstone	5850.7	15.8	31.5	19.5	2701.7	3.3	18,073.0	54.0	54.0	54.1	54.1	54.0	54.0	54.0
Bentonitic shale	5076.1	13.7	27.3	17.5	2344.3	2.9	15,691.3	60.0	60.0	60.3	58.8	60.1	60.1	60.0
Basalt-AZ	4041.8	10.9	21.7	13.9	1866.5	2.3	12,493.4	68.2	68.2	68.4	67.2	68.2	68.2	68.2
IAEA-Th	4700.4	12.7	12.9	16.4	1988.5	2.4	13,753.1			Attenuation (%)				
Standards	3793.7	10.2	12.1	13.2	1629.8	2.0	11,205.0	19.3	19.3	6.1	20.0	18.0	18.0	18.5
Sandstone	2339.2	6.3	6.3	8.2	988.0	1.2	6837.6	50.2	50.2	51.0	50.3	50.3	50.3	50.3
Bentonitic shale	2238.1	6.0	6.0	7.8	945.3	1.2	6542.1	52.4	52.4	53.2	52.4	52.5	52.5	52.4
Basalt-AZ	2009.9	5.4	5.4	7.0	848.9	1.0	5875.1	57.2	57.2	57.9	57.3	57.3	57.3	57.3
IAEA-K	1079.7	2.9	2.9	46.7	584.7	0.7	4403.0		Attenuation (%)					
Standards	734.9	2.0	2.0	30.5	394.6	0.5	2961.5	31.9	31.9	31.1	34.7	32.5	32.5	32.7
Sandstone	685.6	1.9	1.9	28.4	367.4	0.5	2758.3	36.5	36.5	36.5	39.3	37.2	37.2	37.4
Bentonitic shale	642.5	1.7	1.7	25.6	341.2	0.4	2554.4	40.5	40.5	40.5	45.3	41.7	41.7	42.0
Basalt-AZ	582.2	1.6	1.6	23.5	310.2	0.4	2325.1	46.1	46.1	46.1	49.7	46.9	46.9	47.2
	˂370	≤1	≤1	≤1	59	0.07	˂300							

**Table 9:** The Attenuation efficiency (%) of radiological hazard for bentonitic shale, sandstone, and basalt (Basalt-AZ) at 2 cm thickness and standard radionuclide samples NMA-U, IAEA-Th, and IAEA-K.

2 (cm)														
	Ra.eq	Hex	Hin	I γ	D nG/h	AEDE mSv/y	AGDE mSv/y	Ra.eq	Hex	Hin	I γ	D nG/h	AEDR mSv/y	AGDE mSv/y
NMA-U	12,705.1	34.3	68.6	42.5	5869.8	7.2	39,262.0		Attenuation (%)					
Standards	8420.4	22.8	45.5	28.1	3889.8	4.8	26,017.2	33.7	33.7	33.7	34.0	33.7	33.7	33.7
Sandstone	4720.5	12.8	25.4	15.8	2180.0	2.7	14,584.2	62.8	62.8	62.9	62.8	62.9	62.9	62.9
Bentonitic shale	3737.4	10.1	20.0	12.9	1725.4	2.1	11,550.7	70.6	70.6	70.8	69.6	70.6	70.6	70.6
Basalt-AZ	2958.0	8.0	15.8	10.3	1365.8	1.7	9144.6	76.7	76.7	76.9	75.7	76.7	76.7	76.7
IAEA-Th	4700.4	12.7	12.9	16.4	1988.5	2.4	13,753.1		Attenuation (%)					
Standards	3100.9	8.4	9.7	10.8	1329.8	1.6	9148.7	34.0	34.0	24.5	34.6	33.1	33.1	33.5
Sandstone	2138.2	5.8	6.5	7.4	913.1	1.1	6292.0	54.5	54.5	50.0	54.8	54.1	54.1	54.3
Bentonitic shale	1715.6	4.6	4.6	6.3	725.5	0.9	5023.0	63.5	63.5	64.1	61.8	63.5	63.5	63.5
Basalt-AZ	1515.9	4.1	4.1	5.3	640.3	0.8	4431.2	67.7	67.7	68.3	67.8	67.8	67.8	67.8
IAEA-K	1079.7	2.9	2.9	46.7	584.7	0.7	4403.0		Attenuation (%)					
Standards	591.7	1.6	1.6	25.0	318.7	0.4	2396.4	45.2	45.2	45.2	46.4	45.5	45.5	45.6
Sandstone	499.9	1.3	1.3	20.3	266.6	0.3	1998.5	53.7	53.7	53.7	56.7	54.4	54.4	54.6
Bentonitic shale	518.6	1.4	1.4	20.3	274.5	0.3	2053.2	52.0	52.0	52.0	56.5	53.1	53.1	53.4
Basalt-AZ	476.1	1.3	1.3	18.9	252.8	0.3	1892.7	55.9	55.9	55.9	59.5	56.8	56.8	57.0
	˂370	≤1	≤1	≤1	59	0.07	˂300							

## Data Availability

The data presented in this study are available on request from the corresponding author.
